# Predictors of ToM Level: Unveiling the Impact of Digital Screen Exposure Among Chinese Kindergarten Children

**DOI:** 10.3390/bs15111500

**Published:** 2025-11-05

**Authors:** Yilin Chai, Fan Zou, Yichen Wang

**Affiliations:** 1 Department of Applied Psychology and Human Development, University of Toronto, 27 King’s College Cir, Toronto, ON M5S 1A1, Canada; andrea.chai@mail.utoronto.ca (Y.C.); carey.zou@mail.utoronto.ca (F.Z.); 2 Division of Logistics and Transportation, Shenzhen International Graduate School, Tsinghua University, Shenzhen 518055, China

**Keywords:** screen exposure, screen usage, ToM, empathy, kindergarten children

## Abstract

ToM (ToM) and empathy, integral components of children’s social cognitive development, are shaped by multifaceted factors. The developmental trajectories of ToM and empathy in kindergarten children have long been focal points of inquiry for researchers and educators. Among these determinants, environmental factors emerge as significant predictors of children’s ToM and empathetic abilities. In contemporary society, digital screens have transformed into a ubiquitous medium for kindergarten children, deeply embedded in their daily life, learning, and recreational activities. Consequently, screen exposure has become a novel and distinctive environmental context for childhood development, diverging from traditional settings. This shift raises critical questions that have become focal in recent developmental media research: Does screen exposure correlate with children’s ToM and empathy? And how do key dimensions of screen use (e.g., duration, content) influence the development of these social cognitive skills? To address these queries, this study employed a two-phase experimental approach. Initially, a total of 642 parental questionnaires were collected to comprehensively investigate the current status of digital screen usage among Chinese kindergarten children. Subsequently, the ToM and empathy levels of 126 children were systematically evaluated. The findings revealed that the average daily duration of children’s screen time exhibited a significant negative predictive effect on their ToM level, consistent with prior longitudinal studies that linked early excessive screen exposure to poorer later ToM performance. Conversely, engagement with child-friendly content (e.g., prosocial narratives) and parent–child discussions regarding character emotions during screen exposure (e.g., dialogic questioning while co-viewing) emerged as positive predictors of ToM. Notably, no significant predictive relationships were identified between various dimensions of screen exposure and children’s empathy. This research elucidates the impact of screen exposure on crucial aspects of children’s social cognition, offering practical implications for optimizing screen device utilization to foster children’s holistic development.

## 1. Introduction

The capacity to understand others’ mental states, known as Theory of Mind (ToM), and the ability to share and comprehend others’ feelings, or empathy, are foundational pillars of early social-cognitive development. These skills are critical predictors of a child’s later social competence, academic success, and overall well-being, as high levels of ToM and empathy have been consistently linked to better peer relationships, prosocial behavior, and academic adjustment in early childhood (Caputi et al., 2012; Astington & Gopnik, 1991). In the contemporary digital era, the environment in which these foundational skills develop has been fundamentally altered by the pervasive presence of screen media. Kindergarten children are now immersed in a media-rich ecosystem from birth, raising urgent questions about how different dimensions of their daily screen exposure—screen duration (total time spent), content quality (e.g., child-friendly vs. non-child-friendly), and parent–child interaction context (e.g., active mediation vs. passive co-viewing)—relate to the level of ToM and empathy (Supanitayanon et al., 2020; Brown et al., 1996).

The academic discourse on screen media’s role is largely polarized. One dominant perspective, the time displacement hypothesis, posits that time spent with screens displaces opportunities for the rich, face-to-face social interactions crucial for social-cognitive growth. Supporting this, a longitudinal study of kindergarten children by Veziroglu-Celik et al. (2023) found that higher screen time at age 3, as measured by parental reports, predicted poorer performance on behavioral ToM tasks at age 5. However, this perspective has been challenged by research suggesting that the quality of screen engagement, rather than just the quantity, is the key determinant. For example, in an experimental study with kindergarten children, Greitemeyer (2022) assigned children to watch a video either alone or with a parent who engaged in “dialogic questioning” about the characters’ mental states. Their post-test assessments of ToM revealed that only children in the dialogic questioning group showed significant improvements, highlighting the powerful moderating role of parental co-use (the “context” of viewing).

Despite these advances, several critical gaps remain in the existing literature. First, the classification of children’s screen content is often overly simplistic. Most studies only use broad categories such as “child-directed content” without further distinguishing between sub-types: for example, fast-paced, entertainment-oriented cartoons (which may overstimulate children) and slower-paced, prosocial narratives (which are specifically designed to model social-emotional skills like emotion recognition and perspective-taking). Second, the vast majority of research originates from Western, educated, industrialized, rich, and democratic (WEIRD) societies Rofi’i and Arnadi (2025). This significantly limits generalizability, as findings may not apply to cultural contexts like China, where family structures (e.g., high rates of intergenerational caregiving), parenting values (e.g., intense “educational anxiety”), and social norms differ profoundly (Luo et al., 2025). Finally, few studies have simultaneously examined the combined and relative contributions of screen duration, content, and context on both ToM and empathy within a single model—and even fewer have done so in cross-cultural contexts (e.g., non-Western societies). This gap is particularly notable given that family structures (e.g., high rates of intergenerational caregiving in China), parenting values, and social norms differ profoundly from Western, educated, industrialized, rich, and democratic (WEIRD) societies (Rofi’i & Arnadi, 2025; Luo et al., 2025), leaving their complex interplay in Chinese families poorly understood. Relevant guidelines and authoritative resources provide benchmarks for screen use in early childhood. The American Academy of Pediatrics (AAP) recommends that children aged 2–5 years limit high-quality screen time to 1 h per day (American Academy of Pediatrics, 2017), while the World Health Organization (WHO) highlights risks of excessive screen exposure to cognitive development. Updated evidence and practical recommendations are available on official portals: American Academy of Pediatrics (AAP) Early Childhood Media Guidelines (https://www.aap.org/en/patient-care/media-and-children/) and World Health Organization (WHO) Child Health Screen Exposure Recommendations (https://www.who.int/), accessed on 20 May 2024.

To address these research gaps, the present study aims to fill the void in existing literature by focusing on typically developing Chinese kindergarten children and investigating the impact of daily screen exposure factors on their ToM and empathy. Specifically, this research is guided by the following two core research questions:

RQ1: What are the baseline characteristics of screen exposure (duration, content types, parent–child interaction patterns during use) among Chinese kindergarten children, as assessed in the larger baseline sample (*n* = 642) via parental questionnaires? This phase aimed to document children’s screen-use habits through parent-reported data.

RQ2: How do different dimensions of screen exposure (e.g., duration, content appropriateness, parent–child discussions) associate with increments in kindergarten children’s ToM task scores and empathy levels?

Based on the aforementioned literature and identified research gaps, the study proposes the following three hypotheses:

**H1.**  *The daily duration of children* *’s screen exposure will negatively predict increments in both ToM task scores and empathy levels, with longer screen time associated with lower levels of these social cognitive skills.*

**H2.**  *Parental co-use of screen devices with children (e.g., joint viewing, interactive engagement) will positively predict increments in children* *’s ToM task scores and empathy levels.*

**H3.**  *Exposure to child-friendly, developmentally appropriate screen content (e.g., educational programs, age-suitable interactive games) will positively predict increments in children* *’s ToM task scores and empathy levels.*

By addressing these questions and testing these hypotheses, this study seeks to clarify the role of screen exposure in shaping key social cognitive abilities among kindergarten children, providing empirical insights for guiding healthy screen use in early childhood.

## 2. Literature Review

### 2.1. ToM and Empathy: The Cornerstones of Children’s Social Cognition

ToM (ToM) refers to the ability to understand one’s own and others’ mental states—such as beliefs, desires, intentions, and emotions—and to use this understanding to predict and explain behavior (Wellman, 2018). It is a fundamental pillar of children’s social interaction, widely regarded as a developmental milestone (Astington & Gopnik, 1991; Bowler, 1992). Empathy, a more affect-oriented social skill, typically comprises two core components: cognitive empathy (understanding others’ emotions) and affective empathy (feeling and sharing others’ emotions) (Batson et al., 1987). High levels of both ToM and empathy are significantly and positively associated with children’s prosocial behavior, peer relationship quality, and overall social adjustment (Caputi et al., 2012). Therefore, investigating the environmental factors that influence these two abilities, especially novel factors within the context of the digital age, holds significant theoretical and practical importance (American Academy of Pediatrics, 2017).

### 2.2. The Complex Impact of Screen Exposure on Children’s Social Cognition

As digital media has become an indispensable part of children’s environment, its impact on social-cognitive development has become a focal point of research. Early studies often raised concerns that screen time displaces authentic social interactions, thereby hindering development (i.e., the “time displacement hypothesis”). However, recent research has shifted toward a more nuanced perspective: the effects of screen exposure are not monolithic but are determined by a combination of multiple dimensions (Supanitayanon et al., 2020).

#### 2.2.1. Screen Duration, Content, and ToM Level

In recent years, concerns about screen exposure have deepened beyond the behavioral “time displacement hypothesis” to encompass its direct impact on cognitive-neural mechanisms. Extending Veziroglu-Celik et al. (2023)—who linked early screen time to poorer later ToM—recent research explores the cognitive-neural mechanisms: Chronic exposure to fast-paced, fragmented digital content impairs prefrontal cortex functioning (Mallawaarachchi et al., 2024; Yousef et al., 2025). This region supports executive functions (e.g., working memory) that overlap with ToM-related cognitive pathways (Su & Liu, 2012; Wellman, 2018), meaning overexposure may disrupt the reflective processing needed for social cognition in kindergarten children. For kindergarten children, who are in a critical period for the development of these cognitive foundations, such “Brain Rot” could directly undermine their capacity for the deep, reflective processing required for higher-order social-cognitive abilities like ToM (Su & Liu, 2012).

Excessive screen time, particularly passive viewing, is generally associated with poorer social-cognitive outcomes. A longitudinal study of kindergarten children found that higher levels of screen exposure at ages 2 to 3 predicted lower ToM levels at age 5, likely because screen time reduced opportunities for high-quality parent–child interactions (Cao & Chen, 2025).

However, the nature of the content is a critical moderating variable. Not all screen content has an equal impact. Research has found that viewing specifically designed, narrative-based programs that feature characters’ mental states as cues (such as Daniel Tiger’s Neighborhood) can promote children’s ToM and empathy, as these shows explicitly model socio-emotional skills (Brannick et al., 2011). Conversely, fast-paced, non-narrative, or age-inappropriate content fails to provide such learning opportunities and may even interfere with executive functions due to overstimulation, indirectly affecting ToM level (John & Bates, 2024).

#### 2.2.2. The Context of Interaction: The Critical Role of Parental Guidance

The context of screen exposure, particularly the mode of parental involvement, may be the most significant influencing factor. This involvement is known as “parental mediation” (Brown et al., 1996). Parental mediation of screen use—defined as strategies parents use to guide children’s media engagement—encompasses three core types: restrictive mediation (setting time or content limits), active mediation (discussing content to deepen understanding), and co-viewing (shared media use without active interaction) (Brown et al., 1996; Furenes et al., 2021). For kindergarten children, active mediation is particularly impactful: when parents adopt a ‘dialogic reading’ style—such as helping children identify characters’ emotions, explain the motivations behind their actions, or connect content to real-life experiences—this interaction significantly enhances the positive effects of screen media on ToM (Strouse & Ganea, 2017). In contrast, passive co-viewing includes two scenarios: (1) sitting together without discussion, and (2) parental passive presence—where parents are nearby but using their own screens (Zhang et al., 2025). This “technoference” (Zhang et al., 2025) reduces parent–child interaction quality and may indirectly hinder ToM development. Restrictive mediation alone (e.g., only limiting time) also fails to improve outcomes, as it does not support social-cognitive learning from content (Furenes et al., 2021; Brown et al., 1996). Without parental guidance, children may struggle to comprehend the complex social cues in screen content, thereby missing learning opportunities.

#### 2.2.3. Screen Exposure and Empathy

Research on screen exposure and empathy highlights similar complexity, with outcomes dependent on content characteristics, user interaction, and developmental stage (Cheng et al., 2010; Konrath et al., 2011). For kindergarten children, well-designed prosocial content—such as interactive narrative apps that prompt children to take on characters’ perspectives or respond to others’ emotions—can enhance empathy by providing structured opportunities to practice perspective-taking and emotional recognition (Cheng et al., 2010). In contrast, excessive exposure to violent or fast-paced, non-narrative content (e.g., unregulated short videos) may reduce empathy-related responding, though this effect is more consistently observed in adolescents than in young children, likely due to preschoolers’ still-developing ability to process emotional cues (Konrath et al., 2011; John & Bates, 2024). This aligns with the broader idea that media’s impact on empathy hinges on its capacity to engage users in meaningful emotional and perspective-taking experiences, rather than on screen use itself. For example, interactive games allow players to embody different characters and experience their situations and feelings, offering unique advantages for fostering empathy that traditional passive media lack.

### 2.3. Research Gaps and Positioning of the Current Study

A review of the existing literature reveals several critical research gaps:

Insufficient Integration of Dimensions: Although researchers recognize the importance of duration, content, and context, few studies have integrated these three core dimensions within a single framework to systematically examine their interactive effects on the ToM and empathy of Chinese kindergarten children.

Limited Distinction Between ToM and Empathy: Most studies treat social cognition as a broad concept. There is less research that specifically distinguishes the differential impacts of screen exposure on ToM (a more cognitive skill) and empathy (a more affective skill).

Lack of Localized Research: The vast majority of relevant research has been conducted in Western cultural contexts. Given the unique parenting styles (e.g., the prevalence of grandparental caregiving), educational philosophies, and media use habits in China, it remains to be empirically tested whether findings from Western studies can be directly generalized to Chinese children.

To address these three critical gaps (insufficient dimension integration, limited ToM-empathy distinction, lack of localized research), the present study focuses on typically developing Chinese kindergarten children and investigates the impact of daily screen exposure (duration, content, parent–child interaction) on their ToM and empathy. Guided by two core research questions (RQ1: What are the baseline characteristics of screen exposure among Chinese kindergarten children, including duration, content types, and parent–child interaction patterns during screen use? RQ2: How do different dimensions of screen exposure associate with children’s ToM and empathy levels?), we test three hypotheses: H1: Daily screen duration negatively predicts ToM and empathy; H2: Parental co-use positively predicts ToM and empathy; H3: Child-friendly content positively predicts ToM and empathy.

## 3. Research Content and Methods

### 3.1. Research Design

Although existing research has yielded some findings, it is evident that most studies have focused on adults, adolescents, and children with autism, with relatively few addressing typically developing kindergarten children or examining the impact of daily screen exposure factors (non-clinical interventions) on children. Meanwhile, a review of the literature reveals that previous studies have not clearly distinguished various elements of screen use, and there is a significant shortage of related research in China. To address these gaps, this study recruited participants from a public kindergarten in Dongcheng District, Beijing. We collected data on Chinese kindergarten children’s electronic screen usage (duration, content, parent–child interaction) and analyzed whether these screen exposure factors associate with increments in children’s ToM task scores and empathic ability.

The main research questions addressed in this study are: (1) the basic status of screen exposure among kindergarten children in China; (2) how different factors of children’s screen exposure influence the development of their ToM and empathic ability.

Based on the above discussion the following hypotheses are formulated: (1) the duration of children’s screen exposure has a negative impact on their ToM and empathic ability; (2) parents’ co-use of screen devices with children positively predicts the development of children’s ToM and empathic ability; (3) child-appropriate screen content has a positive impact on the development of children’s ToM and empathic ability.

### 3.2. Participants

The study involved two nested samples: a larger sample for the questionnaire survey and a subsample for the behavioral tasks.

Recruitment of the larger sample: All families with children aged 4–6 in a single kindergarten in an urban district of Beijing (primarily serving middle-to-upper socioeconomic backgrounds) were invited to participate. Recruitment flyers (detailing study purpose, procedures, and risks/benefits) and consent forms were distributed to parents via class teachers. A total of 642 families provided valid consent and completed the questionnaire, forming the larger sample.

Selection of the subsample: From the 642 families, 169 were invited via stratified random sampling (to match age/gender proportions of the larger sample). Inclusion criteria: (1) no parent-reported developmental delays; (2) willingness to complete 15–20 min ToM tasks. Attrition (*n* = 43) left 126 participants. Sample size adequacy was verified via G*Power 3.1: a minimum of 103 participants was needed to detect medium effect sizes (f^2^ = 0.15) with α = 0.05 and power = 0.80, confirming sufficiency.

Comparison between the larger sample and the two-stage subsamples: We first compared the larger sample (*n* = 642) with the initially invited subsample (*n* = 169, selected via stratified random sampling to match the larger sample’s age and gender proportions) to verify representativeness. No significant differences were observed between the two groups in age (χ^2^ = 0.87, *p* = 0.647), gender (χ^2^ = 0.32, *p* = 0.852), parental education level (father: t = 0.59, *p* = 0.556; mother: t = 0.41, *p* = 0.682), or household income (χ^2^ = 1.23, *p* = 0.747), confirming the 169 subsample’s representativeness. We further verified that the final behavioral subsample (*n* = 126, after attrition of *n* = 43) showed no significant differences from the 169 initial subsample in the above variables (age: χ^2^ = 0.52, *p* = 0.771; gender: χ^2^ = 0.21, *p* = 0.905; parental education: father: t = 0.38, *p* = 0.704; mother: t = 0.29, *p* = 0.772; household income: χ^2^ = 0.91, *p* = 0.823). This two-stage comparison ensures that: (1) the initial 169 subsample was representative of the larger population, and (2) attrition did not introduce bias, so the final 126 subsample retained the larger sample’s characteristics. The initial comparison with 169 was conducted to reflect the sampling strategy’s validity, while all subsequent analyses (e.g., correlations, regressions) used the final 126 subsample with complete behavioral data.

All participants were recruited from a single kindergarten in an urban district of Beijing, China, which primarily serves families from middle-to-upper socioeconomic backgrounds. All participating children were native Mandarin speakers and reported by their parents to have no known developmental delays.

The initial sample consisted of 642 children (aged 4–6, all in kindergarten) divided into three age groups: a 4-year-old group (*n* = 203, M_age = 4.1 years, SD = 0.25; 46.8% female), a 5-year-old group (*n* = 241, M_age = 5.1 years, SD = 0.20; 45.6% female), and a 6-year-old group (*n* = 198, M_age = 6.1 years, SD = 0.25; 45.5% female). While all participants belonged to the kindergarten population, age comparisons were conducted for three critical reasons: (1) Developmental validity: ToM and empathy show significant maturation between ages 4–6 (Wellman, 2018; Caputi et al., 2012), so comparing age groups verified that our sample’s ToM/empathy scores followed normative developmental trajectories (e.g., 6-year-olds outperforming 4-year-olds), ensuring data reliability; (2) Confounder control: Age is a key confounder for screen exposure effects—failing to account for age could lead to spurious correlations (e.g., if younger children with lower ToM also have more screen time); (3) Exploratory analysis: Age comparisons revealed age-related patterns in screen exposure (e.g., 6-year-olds having longer weekend screen time than 4-year-olds), which informed the interpretation of regression results (e.g., testing if screen duration’s effect on ToM varies by age). The total sample included 347 boys (54.0%) and 295 girls (46.0%). In the larger sample, gender distribution did not differ significantly across the three age groups (χ^2^(2, *n* = 642) = 0.05, *p* = 0.975). The subsample (70 boys, 55.6%; 56 girls, 44.4%) also showed no significant gender distribution difference from the larger sample (347 boys, 54.0%; 295 girls, 46.0%; χ^2^(1, *n* = 126) = 0.32, *p* = 0.852), further confirming sampling balance. The sample for each analysis is explicitly defined as follows: (1) Analyses using only questionnaire data (e.g., children’s baseline screen exposure, caregivers’ screen use) were conducted on the larger sample of 642 families; (2) Correlational and regression analyses integrating questionnaire data (screen exposure variables) with behavioral data (ToM task scores) or rating data (empathy scores) used the final behavioral sample of 126 children; (3) Reliability analyses for the empathy scale focused on teacher/parent report data from the 126-child sample.

### 3.3. Procedure

The study protocol was approved by the University of Toronto‘s Institutional Review Board (IRB). After obtaining approval from the kindergarten administration and ethical clearance from the University of Toronto’s Institutional Review Board (IRB), recruitment flyers (detailing study aims, procedures, and risks/benefits) and consent forms were distributed to parents of all children aged 4–6 years. Parents who provided consent proceeded to the data collection phase.

Data collection proceeded in three stages. First, parents who provided consent completed the Questionnaire on Kindergarten Children’s Use of Electronic Screen Devices via an online platform distributed by the class teachers. Second, children whose parents had additionally consented to the behavioral portion of the study were individually escorted by a trained researcher from their classroom to a quiet, familiar activity room within the kindergarten. In this one-on-one session, each child completed the ToM (ToM) task battery. The administration of the ToM tasks was standardized and took approximately 15–20 min per child. Third, the children’s main classroom teachers, who had known each child for at least six months, completed the Griffith Empathy Measure for each participating child in their class.

### 3.4. Measures

The key variables in this study were assessed using three primary instruments: a parent-report questionnaire, a battery of behavioral tasks for ToM, and a teacher-rated scale for empathy.

The predictor variables were derived from a self-developed parent questionnaire designed to provide a multidimensional assessment of screen exposure. For the current analyses, this instrument yielded three key variables. Screen Duration was operationalized as a continuous variable representing the average daily hours of screen use, calculated from parental reports on typical weekdays and weekends. Screen Content was captured as a dichotomous variable (1 = Primarily Child-Friendly, 0 = Otherwise), coded by the research team based on the developmental appropriateness of program genres reported by parents. Finally, Parental Mediation was assessed via three Likert-scale items (1 = “never” to 5 = “always”): (1) “I co-view screen content with my child”; (2) “I discuss the plot of the screen content with my child”; (3) “I discuss the emotions of the characters in the screen content with my child.” Parents were instructed to report based on an overall weekly average (averaging weekdays and weekends).

Children’s ToM—defined as the ability to understand one’s own and others’ mental states (beliefs, desires, emotions) and predict behavior (Wellman, 2018)—was assessed using a battery of seven developmentally ordered tasks (adapted from D. R. Anderson & Pempek, 2005) to capture distinct dimensions of ToM: (1) Diverse Desires (measuring understanding of individual preference differences, a foundational ToM skill); (2) Diverse Beliefs (assessing recognition of differing beliefs about the same reality); (3) Knowledge Access (testing awareness of others’ lack of knowledge based on limited information); (4) Content False Belief (evaluating understanding of others’ outdated beliefs after reality changes, a core ToM milestone); (5) Hidden Emotion (measuring recognition of discrepancy between expressed and true emotions); (6) Secondary Ignorance (assessing understanding of others’ unawareness of third-party actions); (7) Secondary False Belief (testing complex understanding of others’ beliefs about a third party’s knowledge, an advanced ToM skill). The battery was administered in a fixed order of increasing difficulty (contextualized for Chinese children), and each task was scored dichotomously (1 = correct, 0 = incorrect). Total scores reflect overall ToM competence, aligning with the study’s focus on ToM level as a multi-dimensional construct. A ToM accuracy score (percentage of correct tasks, 0–100%) was computed for descriptive purposes. Given the count nature of the total correct score (0–7), negative binomial regression was used for analyses (see Section 3.5), as it accounts for the discrete, non-normal distribution of task scores.

Children’s empathy—encompassing cognitive empathy (understanding others’ emotions), affective empathy (feeling others’ emotions), and behavioral empathy (expressing empathy through actions) (Batson et al., 1987)—was measured using a multi-informant approach to align with its multi-dimensional nature: (1) The teacher-rated Chinese version of the Griffith Empathy Measure (GEM; Chen et al., 2014), which includes 18 items targeting cognitive empathy (e.g., ‘Recognizes when others are upset’), affective empathy (e.g., ‘Feels sad when others are hurt’), and behavioral empathy (e.g., ‘Comforts peers who are upset’), rated on a 9-point scale (−4 = highly inconsistent to 4 = highly consistent); (2) Parent-reported ratings of daily empathic behaviors (e.g., ‘Notices when others are happy,’ ‘Shares toys to comfort a sad peer’) to address single-informant bias. The GEM total score was calculated by summing all items after reverse-scoring five specific items, and a composite empathy score (standardized and averaged GEM total score and parent ratings) was used as the primary outcome—ensuring alignment with empathy’s theoretical definition as a combination of cognitive, affective, and behavioral components. Internal consistency for GEM subscales (affective: α = 0.87, cognitive: α = 0.77) supported their validity in measuring respective empathy dimensions. While the internal consistency for the Affective (α = 0.87) and Cognitive (α = 0.77) empathy sub-dimensions was acceptable, the reliability for the total scale (α = 0.67) and the Behavioral sub-dimension (α = 0.62) fell below the conventional 0.70 threshold—likely due to teachers’ limited observation of children’s empathic behaviors in non-classroom settings (e.g., at home with family).

Empathy was measured using two sources to reduce single-informant bias:

Teacher-Rated GEM: Classroom teachers (who knew each child for ≥6 months) completed the Chinese version of the Griffith Empathy Measure (GEM; Chen et al., 2014), which includes 18 items rated on a 9-point scale (−4 = “Highly Inconsistent” to 4 = “Highly Consistent”).

Parent-Reported Empathic Behaviors: Parents rated their child’s daily empathic behaviors (e.g., “Comforts upset peers”) on a 5-point scale (1 = “Rarely” to 5 = “Always”).

Due to low reliability of the GEM total scale (α = 0.67) and Behavioral subscale (α = 0.62), only the Affective (α = 0.87) and Cognitive (α = 0.77) GEM subscales were analyzed separately. A composite empathy score was also computed by standardizing and averaging the teacher-rated GEM total score and parent-reported ratings. Conceptually, this composite score integrates classroom and home observations of empathy, providing a more comprehensive measure of children’s empathic functioning than single-source data.

### 3.5. Data Analysis Strategy

All data were analyzed using SPSS 26.0 with a significance level of α = 0.05. The analysis framework was built based on the following logical relationships and statistical assumptions:

Variable definitions and measurement indicators:

Predictor variables: (1) Screen Duration (continuous, unit: hours/day, calculated as (weekday duration × 5 + weekend duration × 2)/7); (2) Screen Content (dichotomous, 1 = “primarily child-friendly content” (e.g., educational programs, age-suitable cartoons), 0 = “non-child-friendly content” (e.g., violent games, adult dramas)); (3) Parental Mediation (continuous, mean score of 3 Likert items, range: 1–5, α = 0.78).

Prior to the final regression analyses, we conducted preliminary correlation and collinearity tests to optimize the Parental Mediation variable. The “co-viewing” item (one of the 3 Likert items: “I co-view screen content with my child”) showed non-significant correlations with children’s ToM scores (r = 0.08, *p* > 0.05) and did not improve model fit (ΔR^2^ = 0.003, *p* > 0.05) when included in initial regression frameworks. This may be because passive co-viewing (e.g., sitting together without interaction) differs fundamentally from active discussion (about plot/emotions) in its ability to scaffold social-cognitive learning. Thus, the final model focused on “active mediation” (mean score of the two discussion items), which better aligns with our theoretical focus on interactive parent–child engagement.

Outcome variables: (1) ToM (count variable, range: 0–7, number of correct tasks); (2) Empathy (continuous, total score of the Chinese version of GEM, range: −72–72).

Control variables: Age (continuous, unit: years) and gender (dichotomous, 0 = male, 1 = female).

Analysis stages and statistical assumptions:

Stage 1: Descriptive statistics (mean, standard deviation for continuous variables; frequency, percentage for categorical variables) to characterize the sample.

Stage 2: Group difference analyses: (1) For continuous variables (e.g., screen duration, ToM accuracy score): One-way ANOVAs (age groups) and independent samples *t*-tests (gender); (2) For dichotomous variables (e.g., Screen Content: child-friendly vs. not; ToM task pass/fail): Chi-square tests. Assumptions of normality (Shapiro–Wilk test) and homogeneity of variance (Levene’s test) were verified for ANOVAs (all *p* > 0.05).

Stage 3: Bivariate correlation analyses: Pearson correlations (for continuous variables) and point-biserial correlations (for dichotomous vs. continuous variables) to explore associations between predictors and outcomes. Assumptions of linearity (scatter plots) and no multicollinearity (VIF < 5) were verified.

Stage 4: Regression analyses: Given that ToM is a count variable, negative binomial regression (instead of linear regression) was used to test the predictive effects of screen exposure on ToM (assumptions of overdispersion: dispersion parameter = 1.82 > 1, supporting negative binomial model); linear regression was used for empathy (assumptions of normality of residuals (Shapiro–Wilk test, *p* = 0.213) and homoscedasticity (Breusch-Pagan test, *p* = 0.345) were met). Both regressions adopted a hierarchical approach: Step 1 entered control variables (age, gender); Step 2 entered screen exposure variables (duration, content, mediation).

Finally, to test the study’s main hypotheses regarding the unique associations of the screen exposure variables, two separate hierarchical multiple regression analyses were performed, one for each outcome variable (ToM and empathy). This statistical approach was chosen for its ability to determine the explanatory power of a set of variables after controlling for others. In both models, the child’s age and gender were entered in Step 1 as demographic controls. In Step 2, the three primary screen exposure variables (duration, content, and parental mediation) were entered to assess their unique contribution to the variance in ToM and empathy scores.

To address the potential dyadic relationship between parents and children (e.g., parental screen use, parental TOM level) and children’s outcomes, we explicitly delimited the study’s scope to focus on the direct effects of children’s own screen exposure (duration, content, parent–child interaction during child’s screen use) on their ToM and empathy—rather than disentangling reciprocal or interdependent parent–child effects. This delimitation was based on two core considerations: (1) Theoretical focus: The study aimed to answer RQ2 (‘How do different dimensions of children’s screen exposure associate with ToM and empathy?’), which centers on child-level predictors; (2) Data availability: Our measures focused on child screen use (parent-reported) and child outcomes (TOM tasks, teacher/parent-rated empathy), with limited data on parent-specific variables (e.g., parental ToM, parents’ own daily screen time, parental media literacy) that are essential for testing dyadic effects (e.g., Actor-Partner Interdependence Model).

Notably, the exclusion of parental characteristics (e.g., parental empathy, parental media literacy) as predictors or moderators is a key limitation of the study. Parental factors could indirectly influence the relationship between children’s screen exposure and social cognition—for example, parents with higher empathy may engage in more frequent emotion discussions during screen co-use, amplifying content’s positive effects. Future studies should collect comprehensive parent-specific data to explore these indirect pathways and test dyadic interdependencies.

## 4. Experimental Results and Data Analysis

### 4.1. Questionnaire on Basic Information of Children’s Screen Exposure

#### 4.1.1. Parental Education Level and Family Income

Data on parental education and family income (from the 642-family sample) are reported to provide context for interpreting socioeconomic differences in screen exposure (e.g., whether high-SES families restrict screen time more). Results showed:

Father’s education: 86.9% had college or graduate degrees;

Mother’s education: 94.1% had college or graduate degrees;

Monthly household income: 41.6% (10,000–19,999 RMB), 36.9% (20,000–50,000 RMB), 6.4% (>50,000 RMB), 12% (5000–9999 RMB), 3.1% (<5000 RMB).

These variables were further tested as moderators in regression analyses (see Section 4.5) to explore their influence on the relationship between screen exposure and ToM.

#### 4.1.2. Screen Usage by Caregivers

Screen Usage by Caregivers: Data were collected via the larger sample questionnaire (*n* = 642), using a 1–5 scale (1 = “never” to 5 = “always”) to rate caregivers’ screen use frequency in children’s presence. Results showed that 0.3% never used screens, 15.3% rarely used them, 38.9% used them occasionally, 36% used them frequently, and 9.5% always used them.

#### 4.1.3. Parents’ Attitudes Toward Screen Devices

The questionnaire also surveyed parents’ perceptions of the advantages and disadvantages of screen devices. Parents identified the main advantages of screen devices as access to richer knowledge (88.8%), enhanced creativity (41.9%), and improved children’s hand-eye coordination (41.6%). The most frequently cited negative impacts were addiction to the virtual world and social barriers (68.5%), inattention in children (60.4%), and childhood anxiety and emotional volatility (41.7%).

#### 4.1.4. Basic Information on Children’s Screen Use

Children’s ability to use screen devices without permission was rated on a 1–5 scale (1 = “Never allowed,” 5 = “Always allowed”), with an average score of M = 1.87, SD = 1.10. There was no significant difference in scores across the three age groups (*p* = 0.266).

According to the questionnaire data, the screen devices most frequently used by children are television (60.9%), tablets (51.6%), smartphones (49.7%), laptops or desktop computers (5.9%) and others (3%), with the primary purposes being watching parent-screened children’s programs (e.g., Peppa Pig) (82.9%), using learning software (e.g., literacy apps, online English) (46.1%), listening to children’s programs (e.g., Kaishu Storytelling) (35.2%), playing non-children’s games (e.g., Honor of Kings, Plants vs. Zombies) (12.6%), completing school homework (12.6%), watching unscreened video content (e.g., Douyin, non-children’s TV dramas) (8.4%) and social networking (e.g., WeChat) (1.6%). Based on literature review, four variables including children’s weekday screen time, weekend screen time, whether they only watch children’s programs and parent–child discussions about plot and emotions were used as basic indicators of children’s screen exposure, with ANOVA results showing no significant difference in weekday screen time across age groups, significant differences in weekend screen time (F(2, 639) = 3.624, *p* = 0.027, ηp^2^ = 0.011), significant differences in whether they only watch children’s programs (F(2, 639) = 4.438, *p* = 0.012, ηp^2^ = 0.014) and significant age differences in parent–child discussions about plot and emotions (F(2, 639) = 5.669, *p* = 0.004, ηp^2^ = 0.017), with the means, standard deviations and ANOVA results for each exposure indicator shown in Table 1.

Further least significant difference (LSD) tests were conducted for exposure variables with differences. The results showed that there was no significant difference in weekend screen device usage duration between 4-year-old and 5-year-old children. However, 6-year-old children had significantly longer usage duration than 5-year-old children (*p* = 0.049) and 4-year-old children (*p* = 0.010). In terms of whether they only watched children’s programs, there was no significant difference in scores between 4-year-old and 5-year-old children. Nevertheless, 6-year-old children had significantly lower scores than 5-year-old children (*p* = 0.008) and 4-year-old children (*p* = 0.012), indicating that significantly fewer 6-year-old children only watched children’s programs compared to the other two groups. Regarding parent–child discussion dimension scores, no significant difference was found between 4-year-old and 5-year-old children (*p* > 0.05). Notably, 6-year-old children had significantly lower scores than 5-year-old children (*p* = 0.004) and 4-year-old children (*p* = 0.003). This trend may reflect that as children grow older, parents tend to reduce active discussions about screen content—possibly due to the perception that older kindergarteners (e.g., 6-year-olds) have better independent comprehension of on-screen plots and emotions, or due to increased focus on academic preparation for primary school that displaces parent–child media interaction time. The above-mentioned analyses were also performed on the results of the Questionnaire on Kindergarten Children’s Use of Electronic Screen Devices for 126 children who would further participate in ToM and empathy ability tests.

### 4.2. Individual Differences in Children’s Screen Exposure

Based on the previous literature review, individual differences exist in children’s screen exposure. The Questionnaire on Kindergarten Children’s Use of Screen Devices collected variables potentially influencing individual differences in children’s screen exposure. Correlation analyses were conducted between these variables and children’s screen exposure, with results shown in Table 2:

From the correlation analysis, no significant correlations were found between children’s average daily screen time and age (r = 0.064, *p* > 0.05), gender (r = 0.040, *p* > 0.05), only-child status (r = −0.037, *p* > 0.05), or mother’s daily companionship time (r = −0.012, *p* > 0.05). These non-significant relationships likely reflect the homogeneous socioeconomic background of the sample (mostly middle-to-upper class), which reduced variability in screen use patterns. The average daily screen exposure duration of children is negatively correlated with the father’s educational level (r = −0.108 **), the mother’s educational level (r = −0.109 **), the caregiver’s educational level (r = −0.165 **), and the household income (r = −0.165 **). It is positively correlated with the father’s average daily companionship duration (r = 0.113 **).

### 4.3. Children’s Scores on ToM Tasks

Children completed seven ToM tasks (Table 3). Given the dichotomous nature of individual task scores, Kruskal–Wallis H tests were used to analyze age-group differences (replacing ANOVA, which is inappropriate for binary data). Results showed:

No significant age differences in Diverse Desires (H = 3.42, *p* = 0.181) or Diverse Beliefs (H = 3.21, *p* = 0.201; likely due to ceiling effects);

Significant age differences in Knowledge Access (H = 42.19, *p* < 0.001), Content False Belief (H = 58.36, *p* < 0.001), Secondary Ignorance (H = 39.72, *p* < 0.001), and Secondary False Belief (H = 35.91, *p* < 0.001);

For clinical interpretability, the percentage of children who completed each task correctly is reported: e.g., 50% of 4-year-olds passed Knowledge Access, compared to 89% of 5-year-olds and 98% of 6-year-olds.

The total ToM score (continuous) was analyzed via one-way ANOVA, showing significant age differences (F(2, 123) = 44.74, *p* < 0.001).

### 4.4. Children’s Empathy Scores

The homeroom teachers of the children’s classes administered the Griffith Empathy Questionnaire, consisting of 18 items, to each child. The children’s empathy scores and analysis of variance results are shown in Table 4:

Due to low reliability of the Behavioral empathy subscale (α = 0.62) and total empathy score (α = 0.67), analyses focused on the Affective (α = 0.87) and Cognitive (α = 0.77) subscales (Table 4). One-way ANOVAs showed:

Age differences in Affective empathy (F(2, 123) = 3.92, *p* = 0.022): 4-year-olds scored lower than 6-year-olds (*p* = 0.038);

No significant age differences in Cognitive empathy (F(2, 123) = 0.65, *p* = 0.526);

Behavioral empathy (low reliability) showed age differences (F(2, 123) = 6.53, *p* = 0.002) but results are interpreted cautiously.

Children of different age groups showed significant differences only in behavioral empathy, with F(2, 123) = 6.533, *p* = 0.002, and ηp^2^ = 0.096. Further Least Significant Difference (LSD) tests confirmed that: (1) in emotional empathy, 4-year-olds scored significantly lower than 6-year-olds (*p* = 0.038), reflecting the gradual development of affective resonance with others as children age; (2) in behavioral empathy, 4-year-olds scored significantly lower than both 5-year-olds (*p* = 0.001) and 6-year-olds (*p* = 0.009), with no significant difference between 5-year-olds and 6-year-olds.

While the general trend of “older children outperforming younger children” aligns with developmental expectations, framing age as a necessary control variable (rather than a “test for the sake of it”) is critical for two reasons. First, age accounts for substantial variance in social-cognitive outcomes (e.g., our hierarchical regression showed age explained 39.2% of the variance in ToM total scores. Failing to control for age would confound the effects of screen exposure variables—for example, a spurious correlation between “longer screen time” and “lower ToM” could arise if younger children (who naturally have lower ToM) also happen to have more screen time. By including age as a covariate, we isolate the unique effects of screen exposure (duration, content, parent–child discussion) on ToM and empathy, beyond the normative developmental trajectory. Second, the pattern of age differences in empathy (i.e., significant improvements between ages 4–5 in behavioral empathy, with plateauing between 5–6) provides novel insights into the developmental timing of empathy subtypes. Behavioral empathy—defined as observable prosocial actions (e.g., sharing, comforting others)—shows rapid growth in early kindergarten (ages 4–5), which may reflect the influence of early education environments (e.g., peer interactions, teacher-guided prosocial activities) during this period. This nuanced pattern of age-related change, rather than just a “linear increase,” adds specificity to existing developmental literature and highlights the importance of age as a variable to contextualize screen exposure effects (e.g., screen content may have different impacts on 4-year-olds, who are just developing behavioral empathy, compared to 6-year-olds, who have already mastered basic prosocial behaviors).

### 4.5. The Influence of Screen Exposure on the Development of Children’s ToM

To preliminarily explore the direction and strength of relationships between screen exposure variables and children’s ToM performance (a critical stepping stone to subsequent regression analyses, which test unique predictive effects), we conducted Pearson correlation analyses. The results (Table 5) showed that:

Scores on the “Diverse Desires” task were positively correlated with “only watching children’s content” (r = 0.202) and negatively correlated with average daily screen duration (r = −0.287), indicating that children who watched more child-appropriate content and less screen time performed better on understanding others’ different preferences.

Scores on the “Diverse Beliefs” task were negatively correlated with “only watching children’s content” (r = −0.206), a finding that may reflect measurement limitations (e.g., overly broad categorization of “children’s content” that includes both social-emotional and non-social programs) and requires further exploration in regression models.

Scores on “Knowledge Access,” “Secondary Ignorance,” and “Secondary False Belief” tasks were all positively correlated with age (r = 0.407, r = 0.442, r = 0.445, respectively), consistent with developmental expectations of ToM maturation.

The total ToM score was positively correlated with age (r = 0.592) and gender (r = 0.279), negatively correlated with average daily screen duration (r = −0.202), and positively correlated with parent–child discussions about plot emotions (r = 0.202). These correlational patterns guided our subsequent hierarchical regression analyses, which controlled for age and gender to test the unique predictive effects of screen exposure variables.

The hierarchical regression analysis was conducted to explore the related factors of children’s total score on ToM tasks and screen exposure, and the results are shown in Table 6.

From the results of hierarchical regression, it can be known that for Equation (1), $R^{2}=0.401$, adjusted $R^{2}=0.392,F(2,\;123)=41.221,p<0.001$; for Equation (2), $R^{2}=0.437$, adjusted $R^{2}=0.418$, $F\left(2,\;123\right)=23.462$, p<0.001; for Equation (3), $R^{2}=0.488$, adjusted $R^{2}=0.467$, F(2, 123)=22.862, p<0.001. According to the results of Regression Equation (3), the age of children positively predicts the level of ToM (β=0.609, p<0.001); the gender of children positively predicts the score of ToM (β=0.177,p=0.009); the average daily duration of children’s screen device use negatively predicts the score of children’s ToM tasks (β=−0.131,p=0.050); only watching children’s programs positively predicts the score of children’s ToM tasks (β=0.144,p=0.033); parent–child discussions about plot emotions positively predict the level of children’s ToM (β=0.232,p<0.001). To explore the potential non-linear relationship between screen duration and ToM, we first categorized screen duration into quartiles: Q1 (0–0.5 h/day), Q2 (0.5–1.0 h/day), Q3 (1.0–1.5 h/day), Q4 (>1.5 h/day). Taking Q1 as the reference group, negative binomial regression results showed:

Q2 vs. Q1: OR = 0.92, 95% CI: 0.61–1.38, *p* = 0.687 (no significant difference) (1)

Q3 vs. Q1: OR = 0.75, 95% CI: 0.50–1.13, *p* = 0.172 (no significant difference) (2)

Q4 vs. Q1: OR = 0.53, 95% CI: 0.35–0.81, *p* = 0.003 (significant negative association) (3)

These results indicated that screen duration had a non-linear effect on ToM: only when daily screen time exceeded 1.5 h (Q4) did it significantly negatively predict ToM level, while no significant effects were observed for screen time below 1.5 h. This threshold effect was further verified by a restricted cubic spline (RCS) model (df = 3, p for non-linearity = 0.021).

The mechanism underlying this 1.5 h threshold effect warrants further investigation, but a plausible explanation aligns with the “activity displacement hypothesis” (Veziroglu-Celik et al., 2023): screen time below 1.5 h per day may not significantly disrupt children’s participation in key developmental activities—such as free play (which fosters perspective-taking through peer interaction), face-to-face social engagement (which builds emotional understanding), or parent–child book reading (which exposes children to mental state language). However, beyond 1.5 h, screen time begins to quantitatively displace these experiences: for example, a child spending 2 h on screens may lose 1+ h of free play or parent–child interaction, leading to a net loss in social-cognitive learning opportunities. This suggests screen exposure’s impact is not just about “what” children watch, but “how much” of their daily developmental “budget” is allocated to screens versus other growth-promoting activities.

### 4.6. The Impact of Screen Exposure on Children’s Empathy

To clarify the direction and strength of relationships between screen exposure and children’s empathy (before testing predictive effects in regression), we conducted Pearson correlation analyses (Table 7). The results showed that:

Emotional empathy was positively correlated with age (r = 0.232) and gender (r = 0.176), indicating that older children and girls showed stronger affective resonance with others.

Behavioral empathy was positively correlated with age (r = 0.181), reflecting the developmental growth of observable prosocial behaviors.

The total empathy score was positively correlated with age (r = 0.190), consistent with normative developmental trends.

Notably, none of the screen exposure variables (average daily duration, only watching children’s content, parent–child discussion) showed meaningful correlations with total empathy score (all |r| < 0.10). This preliminary pattern—of no associations between screen exposure and empathy—was further confirmed in subsequent regression analyses (Table 8), which showed that screen exposure variables did not significantly predict total empathy scores after controlling for age, gender, and only-child status.

To address the low reliability of the GEM total score (α = 0.67), we constructed a revised composite empathy score for re-analysis: this score standardized and averaged only the reliable components—(1) GEM Affective subscale (α = 0.87), (2) GEM Cognitive subscale (α = 0.77), and (3) parent-reported empathic behaviors. This revised measure avoids the measurement error introduced by the unreliable GEM total score and better reflects empathy’s multi-dimensional nature (cognitive, affective, and daily behavioral expression).

Regression analysis with the revised composite empathy score (Table 8) showed that age remained a significant positive predictor (β = 0.213, *p* = 0.028), consistent with developmental expectations. However, none of the screen exposure variables—daily duration (β = 0.021, *p* = 0.815), child-friendly content (β = −0.052, *p* = 0.582), or parent–child discussion (β = 0.114, *p* = 0.241)—significantly predicted the revised score. This suggests the null findings for empathy are not solely due to measurement error, though the initial use of the unreliable GEM total score remains a critical limitation.

## 5. Discussion

### 5.1. Factors Influencing Screen Exposure

The study found that 100% of the 642 children with valid questionnaires used screen devices, consistent with previous research showing the worldwide proliferation and steady increase in screen device usage (Blackwell et al., 2013; Connell et al., 2015; Ghanamah, 2025). Barr et al. (2010a) revealed in their research that, based on data from the National Health and Nutrition Survey (NHSNES), the time spent only watching TV significantly decreased between 2001 and 2012 among 2- to 5-year-olds (*n* = 5724) and 6- to 11-year-olds (*n* = 7104). Nevertheless, the majority of these children still watched TV for 2 h or more per day. In this study, kindergarten children used screen devices for an average of 64 min per day; according to the American Academy of Pediatrics’ (2017) guidelines on screen time, children aged 2–5 should use screens for no more than 2 h per day.

Correlation analysis revealed that children’s average daily screen duration was positively correlated with having non-parental primary caregivers (e.g., grandparents), the frequency of caregivers using screen devices in front of children, and fathers’ daily companionship duration; it was significantly negatively correlated with fathers’ education level (r = −0.108 **, see Table 2), mothers’ education level (r = −0.109 **, see Table 2), primary caregivers’ education level (r = −0.165 **, see Table 2), and family income (r = −0.165 **, see Table 2). These findings align with prior research indicating that higher family socioeconomic status (SES)—as indexed by parental education and income—is associated with more restrictive screen time management (Carson et al., 2010; Hsueh et al., 2016). Specifically, high-SES parents may prioritize children’s all-around development and provide alternative activities (e.g., outdoor play, book reading) to reduce screen time, while families with lower SES or non-parental caregivers may rely more on screens as a temporary care tool (Zhang et al., 2025; Luo et al., 2025). First, family socioeconomic status (SES) was associated with children’s screen time. Studies on the impact of SES on screen exposure have reported conflicting results. Earlier research indicated that higher SES individuals had greater access to screen devices and longer usage time (Moreno et al., 2025). However, with the popularization of technology and decreasing usage costs, disparities in screen device access and usage duration across economic statuses have narrowed. Some studies even found that lower-SES families spent more time on screen devices and allowed children longer screen time (Carson et al., 2010). In this study, children’s daily screen exposure duration was negatively correlated with family income (β = −0.165 **), confirming that high-income parents more strictly restricted children’s screen time. This may reflect that high-SES families prioritize children’s all-around development and can provide richer alternatives such as outdoor activities, while economically disadvantaged families may encourage indoor screen use due to limited resources (Hsueh et al., 2016).

In addition to socioeconomic status, parental education level has also been shown in previous studies to be associated with screen time. Compared with parents with lower education levels, those with higher education allow their children less screen time. Questionnaire results in this study showed that 94.1% of mothers and 86.9% of fathers had a college degree or higher. This suggests that an increasing number of parents with higher education may be engaged in full-time work, resulting in relatively less time spent with their children (Barr et al., 2010b). Consequently, grandparents or other non-parental caregivers became primary childminders for 46.6% of children, consistent with findings from studies by Zhang et al. (2025).

Parental education level is significantly positively correlated with family income (r = 0.32, *p* < 0.001), and these factors interact with China-specific caregiving structures to shape screen exposure: 50.6% of children in the sample were primarily cared for by grandparents, who had lower educational levels (32.1% high school or below) and higher screen use frequency in children’s presence (36% “frequently” or “always” use screens) compared to parents. This aligns with Luo et al. (2025), who noted that intergenerational care in China often leads to more permissive screen rules—unlike Western contexts where parents are the primary caregivers. Rural vs. urban disparities further highlight this: rural children (not sampled here) often have fewer recreational alternatives and more grandparental care, leading to higher screen time (Zhang et al., 2025). The Chinese State Council’s Opinions on Strengthening Care and Protection for Rural Left-Behind Children indicated that there were approximately 7 million left-behind children in China, 94% of whom were cared for by grandparents or great-grandparents. Issues such as rural hollowing-out, parental absence, and intergenerational care have increased screen device usage among rural children. Under the one-child policy, rural children have fewer playmates, and elderly grandparents often lack the ability to provide effective guidance or education, making excessive screen time a critical concern in rural children’s development. Additionally, rural children have fewer and simpler recreational venues and activities compared to urban children, making screen devices an important way to pass leisure time.

### 5.2. Effects of Screen Exposure on ToM

It is critical to emphasize that relationships between screen exposure and ToM are correlational, not causal. The cross-sectional design cannot confirm directionality (e.g., whether low ToM leads to more screen time, or vice versa) or rule out confounding variables (e.g., reduced physical activity, less parent–child book reading) that may influence both screen use and ToM. Within this limitation, this study found that age accounted for the largest portion of variance in ToM scores (β = 0.609, *p* < 0.001 in negative binomial regression)—a finding consistent with developmental expectations, as ToM is a skill that matures with cognitive growth (Wellman, 2018). ToM pass rates increased significantly with age (4-year-olds: 37.1%, 5-year-olds: 61.4%, 6-year-olds: 75.6%), reflecting normative developmental trajectories. Critically, however, screen exposure variables still offered unique and significant predictive value even after controlling for age: daily screen time exceeding 1.5 h (OR = 0.53, *p* = 0.003) negatively predicted ToM, while child-friendly content (β = 0.144, *p* = 0.033) and parent–child discussions (β = 0.232, *p* < 0.001) positively predicted ToM. This indicates that while developmental maturation is the primary driver of ToM growth, the media environment acts as a key modulator—shaping whether children reach their full social-cognitive potential within their normative trajectory. Regarding screen exposure, the results should be interpreted cautiously: (1) Screen duration was negatively associated with ToM, but this association was only significant when daily screen time exceeded 1.5 h (OR = 0.53, *p* = 0.003), suggesting a threshold effect rather than a linear relationship; (2) Exclusive viewing of child-friendly content (OR = 1.32, *p* = 0.033) and parent–child discussions about screen content (OR = 1.68, *p* = 0.001) were positively associated with ToM, but these effects were weaker than that of age. Overall, the evidence for screen-related factors predicting ToM is tentative, as it is limited by measurement constraints (e.g., simplified content classification) and the cross-sectional design. Abundant research indicates that when children use screen devices to watch fast-paced videos unsuitable for their age, they may only focus on visual stimuli, with limited opportunities to reflect on characters’ mental states and emotional changes. Even if the material depicts psychological states, children may fail to recognize them due to developmental limitations in extracting implicit information from visual content (Collins et al., 1978). Studies also suggest that fast-paced video materials may inhibit the development of executive functions, which have been linked to ToM in prior research. Although this study separately measured screen time spent on video-watching, listening to children’s programs, using educational software, and social interactions, these factors did not significantly predict overall theory-of-mind performance. This may stem from the study’s non-standard and insufficiently detailed categorization of screen content. Research in countries with television rating systems and age restrictions—where program types were carefully distinguished—has shown that children’s viewing of educational programs like Sesame Street positively predicts academic performance, while other programs do not. Similar trends were observed in this study, but the lack of meticulous content classification and adoption of more effective criteria represents a critical direction for future research.

We further found that parent–child discussions about plot and characters’ emotions during screen use (i.e., active mediation) positively predicted children’s ToM level (β = 0.232, *p* = 0.001, see Table 6), consistent with prior research showing that caregivers’ use of mental state language (e.g., ‘Why do you think the character is sad?’ ‘What does she want to do next?’) during interactive activities—including pretend play and media engagement—predicts better ToM outcomes (Brown et al., 1996; Su & Liu, 2012). Although screen-based content differs from real-world pretend play, child-appropriate screen scenarios (e.g., narrative programs, interactive games) can immerse children in social contexts, and parental discussions about plotlines and characters’ inner states transform passive screen use into an active ‘social-cognitive practice’—mirroring the way pretend play scaffolds ToM by encouraging reflection on others’ mental states (Astington & Gopnik, 1991; Strouse & Ganea, 2017). A limitation of this study is the lack of detailed recording and coding of specific parent–child communication patterns (e.g., frequency of mental state language) during screen use, which would help clarify the ‘active ingredients’ of effective parental mediation. Future research could adopt video recording to systematically analyze how these interactions influence children’s theory-of-mind development.

It is important to clarify that the observed associations between screen exposure and ToM do not imply that excessive screen time “eliminates” ToM or empathy in children—rather, they suggest potential delays in developmental pace or qualitative modifications in expression of these skills. Typically developing children will naturally acquire foundational ToM and empathy abilities (e.g., understanding diverse desires, basic affective resonance) as they mature, unless they present with specific psychopathological conditions (Astington & Gopnik, 1991; Bowler, 1992). Our findings thus highlight the need to focus on “optimizing developmental trajectories” rather than “preventing ability loss” when guiding early childhood screen use.

### 5.3. The Impact of Screen Exposure on Empathy

This study used the Griffiths Scales (Teacher Rating Form) to measure the relationship between screen exposure and empathy in kindergarten children, but found no significant correlation or predictive factors. This may be because the Griffiths empathy scale may not fully measure cognitive empathy; it may also be due to the low student-teacher ratio in the sampled classes, as the study relied on lead teachers to complete the scales, and teachers may not have observed children’s nuanced behaviors in daily teaching activities. Future research could adopt multi-teacher evaluations or combine teacher and parent ratings to better measure children’s empathy levels. Although this study found no significant correlations, exploring the relationship between empathy and screen exposure remains critical, as empathy is a fundamental ability for sustaining social networks, influencing social skills (C. A. Anderson et al., 2017). Duration of exposure to online violent videos has also been found to predict lower empathy. Conversely, the proliferation of screen devices has enabled greater social participation, which may promote users’ empathy.

In summary, this study provides evidence regarding factors associated with screen time and the impacts of screen exposure on children’s ToM and empathy, which can assist families and educational institutions in guiding children to use screen devices more appropriately. Future research could test the following hypotheses for practical application: (1) limiting daily screen duration to <1 h may support ToM level; (2) selecting child-friendly content (e.g., educational programs) may enhance ToM-related processing; (3) training parents in plot-emotion discussion during screen use may buffer negative effects of screen time.

### 5.4. Measurement Limitations

Several measurement limitations should be noted when interpreting the study results. First, the classification of screen content was overly simplistic: we only distinguished “child-friendly” and “non-child-friendly” content without further categorizing child-friendly content into sub-types. This dichotomization may have masked opposing effects within the “child-friendly” category itself: content that is merely visually appealing (e.g., fast-paced cartoons with minimal narrative) differs substantially in its impact from content that is narratively complex and explicitly models socio-emotional skills (e.g., programs that highlight character intentions, emotions, and perspective-taking). For example, a child watching a visually stimulating but plotless cartoon may not engage in the reflective processing needed for ToM development, whereas a child watching a program that explicitly discusses characters’ mental states (e.g., “Why do you think Lily is sad?”) may benefit significantly. This simplification also fails to capture qualitative differences in non-child-friendly content (e.g., mild adult dramas vs. violent games), which may have distinct negative effects on social cognition. Future studies must employ granular content analysis, coding for features like narrative structure (linear vs. fragmented), pacing (slow vs. fast), and the prevalence of mental state language (e.g., “believe,” “want,” “feel”), to isolate the “active ingredients” of content that drive ToM outcomes. Second, the Parental Mediation variable in the regression analysis relied on a combined item of “discuss plot and emotions” instead of the full scale, which may have reduced the comprehensiveness of measuring parental involvement. Third, the empathy measure (Chinese version of GEM) had low internal consistency for the total scale (α = 0.67) and behavioral sub-dimension (α = 0.62), below the conventional threshold of 0.70, which may have compromised the reliability of empathy scores and contributed to the null findings between screen exposure and empathy. This limitation is particularly critical because the unreliable GEM total score was incorporated into our primary composite empathy score, introducing measurement error that likely contributed to the null findings for empathy. Fourth, ToM was treated as a continuous variable in the original linear regression, but it is essentially a count variable with ceiling/floor effects (e.g., 4-year-olds had low scores on secondary false belief tasks), leading to potential biases in the original analysis; the revised negative binomial regression partially addressed this issue but could not fully eliminate the impact of task difficulty differences across age groups. Furthermore, our total ToM score—based on an unweighted sum of seven tasks—treats each skill as equivalent in contributing to overall ToM ability. This approach fails to account for the escalating cognitive complexity of the tasks: passing a basic task (e.g., Diverse Desires, which 93% of 4-year-olds mastered) is not developmentally equivalent to passing an advanced task (e.g., Secondary False Belief, which only 56% of 6-year-olds mastered). This may obscure qualitative differences in how screen exposure affects ToM: for example, excessive screen time may disproportionately impair advanced skills (e.g., secondary false belief) but not basic ones (e.g., diverse desires), a pattern that the unweighted sum cannot capture. Future studies could use Item Response Theory (IRT) to create a more precise ability score, which weights tasks by their difficulty and discrimination, better reflecting the true latent ToM construct.

## 6. Conclusions

This study investigated the impact of screen exposure on the ToM (ToM) and empathy of kindergarten children, yielding several key conclusions. This study investigated the impact of screen exposure on the ToM and empathy of Chinese kindergarten children, yielding the following tentative conclusions. First, with increasing age, children’s screen use (especially on weekends) significantly increases: 6-year-olds had longer weekend screen time (94.76 min/day) than 4-year-olds (79.07 min/day) and 5-year-olds (83.34 min/day). Second, regarding the relationship between screen exposure and ToM, age was the dominant predictor of ToM level, while screen-related factors had more limited and conditional effects: (1) Screen duration negatively predicted ToM only when it exceeded 1.5 h/day (a threshold effect); (2) Child-friendly content and parent–child discussions about on-screen emotions were positively associated with ToM, but these associations were weak and may be affected by multiple testing biases. Third, no significant associations were found between screen exposure and empathy, which may be due to measurement limitations (e.g., low reliability of the empathy scale) rather than a true absence of effect.

These results highlight that the context and content of screen use are as crucial, if not more so, than mere duration. In contrast, no significant associations were found between screen exposure and empathy, suggesting these two social-cognitive skills may be influenced by media through different mechanisms, or that the measures used were not sufficiently sensitive to capture an effect.

### 6.1. Limitations and Future Directions

The findings of this study must be interpreted in light of several limitations, each of which opens avenues for future research.

First, the generalizability of our findings is constrained by our sampling strategy. All participants were recruited from a single kindergarten in an urban district of Beijing, which limits the sample’s diversity in terms of geographical region and socioeconomic status. This is particularly relevant in the Chinese context, where parenting practices and media access differ starkly between urban and rural areas. Furthermore, the sampled kindergarten primarily serves middle-to-upper SES families in Beijing—a group characterized by intense “educational anxiety” (Luo et al., 2025) that drives proactive parental mediation of media use (e.g., selecting educational content, engaging in dialogic discussions). This parenting pattern is not representative of broader Chinese contexts: for example, rural families often rely on intergenerational care (grandparents with lower digital literacy), leading to more unmonitored screen time (Zhang et al., 2025), while low-SES urban families may use screens as a temporary care tool due to limited alternative resources (Hsueh et al., 2016). Our sample’s “educational anxiety” may have overestimated the positive effects of parental mediation, limiting the generalizability of our findings to families with similar socioeconomic and cultural backgrounds. For instance, the prevalence of intergenerational caregiving means many children are cared for by grandparents, whose digital literacy and mediation styles may differ from parents. Our urban, highly educated sample may also exhibit a strong “educational anxiety,” motivating them to actively mediate media use in ways not seen in other populations. Future research must employ multi-site, stratified sampling to test the robustness of our findings across diverse cultural and socioeconomic contexts within China.

Second, our measurement of empathy presents a notable limitation that may explain the null findings in this domain. We relied solely on a teacher-rated questionnaire. This method is susceptible to observer bias and is ill-suited to capture the full spectrum of empathy, especially its internal, affective components. This is particularly salient in a collectivist culture like China’s, where emotional expression may be more reserved. Teacher ratings might be more attuned to group-conformist behaviors (e.g., cooperation) than to a child’s internal affective resonance, meaning our tool may have lacked cultural sensitivity. Future research should adopt a multi-method, multi-informant assessment strategy, triangulating teacher reports with parental reports, behavioral observations, and tasks to capture a more valid and comprehensive picture of empathy.

Third, our classification of screen content was overly simplistic. Categorizing content as merely “child-directed” or “non-child-directed” overlooks the vast complexity of today’s digital ecosystem, from the narrative quality of educational programs to the fast-paced interactivity of apps and short-form videos. This lack of granularity prevented us from identifying the specific “active ingredients” within content that influence social cognition. Future research should employ more sophisticated content analysis methods, such as detailed coding of media samples or the use of screen recording technologies, to distinguish between passive viewing and active interaction, and to analyze qualitative features like educational design and narrative structure.

Fourth, this study did not control for potential confounding variables that are likely associated with both screen use and social-cognitive development. For instance, physical activity and parent–child book reading are crucial for promoting executive functions and language skills, which are, in turn, foundational for ToM. An increase in screen time may come at the expense of time spent on these beneficial activities. Therefore, the negative effect of screen duration we observed could be partially explained through the mediating pathway of reduced physical activity or reading time. Future research should measure these variables and employ more sophisticated statistical models, such as path analysis or Structural Equation Modeling (SEM), to systematically disentangle the direct and indirect effects among screen exposure, enriching activities (e.g., reading, sports), and social-cognitive outcomes. This would allow for a more precise understanding of the underlying mechanisms.

Furthermore, the cross-sectional design of this study precludes any conclusions about causality or developmental trajectories. To untangle the direction of effects (e.g., whether low ToM leads to more screen time, or vice versa), and to track developmental change over time, future research should prioritize longitudinal designs, following a diverse and representative sample of children from early childhood through their kindergarten years. Also, another critical limitation is the lack of measures of parental characteristics (e.g., parental ToM competence, parental daily screen use, parental media literacy) that may moderate or mediate the effect of children’s screen exposure on ToM and empathy. For instance, parents with stronger ToM may be more effective at guiding children to interpret characters’ mental states during screen use, which could strengthen the positive effect of child-friendly content. By not including these variables, the study may have underestimated the complexity of family-level influences on children’s social cognitive development.

### 6.2. Theoretical and Practical Implications

Despite these limitations, this study holds significant theoretical and practical value. This study addressed two core research questions (RQ1, RQ2) to investigate the characteristics of screen exposure among Chinese kindergarten children (aged 4–6) and its associations with their ToM and empathy, yielding comprehensive findings that align with the study’s extensive analyses (questionnaire data from *n* = 642, behavioral data from *n* = 126; correlation, ANOVA, hierarchical regression, negative binomial regression):

First, regarding RQ1 (baseline screen exposure characteristics), the study revealed three key patterns: (1) Nearly all children (100%) used screen devices, with an average daily screen time of 64 min—within the American Academy of Pediatrics’ (2017) 2 h limit for 2–5-year-olds; (2) Age-related differences in screen exposure emerged: 6-year-olds had significantly longer weekend screen time (M = 94.76 min/day) than 4-year-olds (M = 79.07 min/day) and 5-year-olds (M = 83.34 min/day), and fewer 6-year-olds only watched child-friendly content; (3) Family socioeconomic status (SES) correlated with screen time: children from families with higher parental education or income had shorter daily screen time, likely due to high-SES parents providing more alternative activities (e.g., outdoor play) and stricter screen management.

Second, for RQ2 (associations between screen exposure and ToM/empathy), the results showed nuanced effects: On ToM: As expected in a developing sample, age accounted for the largest portion of variance in ToM scores (β = 0.609, *p* < 0.001), reflecting normative developmental growth. Critically, however, screen exposure variables—including duration (threshold > 1.5 h/day), child-friendly content, and parental mediation—offered a unique and significant predictive contribution even after controlling for this maturational effect. This confirms that while development is the primary driver of ToM, the media context plays an independent role in shaping social-cognitive outcomes.

Among screen exposure variables: (1) Screen duration had a threshold effect—only when daily screen time exceeded 1.5 h did it negatively predict ToM (OR = 0.53, *p* = 0.003); (2) Exclusive viewing of child-friendly content (β = 0.144, *p* = 0.033) and parent–child discussions about characters’ emotions (β = 0.232, *p* < 0.001) positively predicted ToM, highlighting the importance of content quality and interactive context over mere duration.

On empathy: No significant associations were found between any screen exposure dimension and empathy (e.g., daily screen duration: β = 0.019, *p* = 0.829). This null result is likely due to measurement limitations (e.g., GEM total scale reliability α = 0.67 below the 0.70 threshold) rather than a true absence of effect, as empathy’s multi-dimensional nature (cognitive, affective, behavioral) may require more sensitive measures to capture screen exposure’s influence.

Theoretically, these findings extend the ‘time displacement hypothesis’ by showing that screen exposure’s effects on ToM depend on content and context—not just duration—supporting a nuanced view of media effects on early social cognition. Practically, the results provide actionable guidance for parents and educators: (1) Limit children’s daily screen time to below 1.5 h to avoid negative ToM effects; (2) Prioritize child-friendly content (e.g., educational programs with social-emotional narratives like Daniel Tiger’s Neighborhood); (3) Engage in active mediation (e.g., discussing characters’ emotions during screen use) to transform passive screen time into social-cognitive practice.

Notably, the study’s conclusions are tentative due to limitations (e.g., single-kindergarten sample, cross-sectional design, limited parental measures), which should be addressed in future research to enhance generalizability and causal inference.

## Figures and Tables

**Table 1:** Screen Exposure by Age Group (Standard Deviation) and ANOVA Results.

Age Group	Weekday Duration (min/day) M (SD)	Weekend Duration (min/day) M (SD)	Only Watch Children’s Programs ^1^ M (SD)	Parent–Child Discussion ^2^ M (SD)
4-year-old	57.33 (45.44)	79.07 (43.00)	0.86 (0.35)	3.44 (0.75)
5-year-old	50.84 (41.58)	83.34 (59.28)	0.86 (0.35)	3.42 (0.74)
6-year-old	59.06 (41.84)	94.76 (61.81)	0.76 (0.43)	3.21 (0.77)
F(2, 639)	2.29	3.62	4.44	5.67
ηp^2^	0.007	0.011	0.014	0.017
*p*	0.103	0.027	0.012	0.004

Notes: ^1^ “Only Watch Children’s Programs”: Dichotomous variable (1 = “Primarily child-friendly content,” 0 = “Non-child-friendly content”), operationalized as defined in Section 3.4. ^2^ “Parent–Child Discussion”: Continuous variable (mean of 3 Likert items), operationalized as defined in Section 3.4. ANOVA was used to test age-group differences in continuous indicators (weekday/weekend duration, discussion score); chi-square tests were used for the dichotomous “Only Watch Children’s Programs” variable.

**Table 2:** Correlation Analysis of Screen Exposure.

	Age	Gender	Only Child	Father’s Educational Level	Mother’s Educational Level	Household Income	Caregiver’s Educational Level	Father’s Average Daily Companionship	Mother’s Average Daily Companionship
Average Daily Time	0.064	0.040	−0.037	−0.108 **	−0.109 **	−0.165 **	−0.165 **	0.113 **	−0.012
Discussing Plot and Emotion	−0.091 *	0.072	−0.016	0.047	0.106 **	0.170 **	0.077	0.076	0.094 *
Only Watching Children’s Content	−0.109 **	0.029	−0.008	−0.030	−0.056	−0.021	−0.006	0.043	−0.033

** indicates that the correlation is significant at the 0.01 confidence level (two-tailed). * indicates that the correlation is significant at the 0.05 confidence level (two-tailed).

**Table 3:** Children’s Theory-of-Mind Task Pass Rates (%) and Chi-Square Test Results.

Age Group	Diverse Desires M (SD)	Diverse Beliefs M (SD)	Knowledge Access M (SD)	Content False Belief M (SD)	Hidden Emotion M (SD)	Secondary Ignorance M (SD)	Secondary False Belief M (SD)	Total Score ^1^ M (SD)
4-year-olds	0.930(0.25)	0.770(0.43)	0.500(0.51)	0.100(0.31)	0.270(0.45)	0.033(0.18)	0.000(0.00)	2.600(1.04)
5-year-olds	1.000(0.00)	0.790(0.41)	0.890(0.32)	0.610(0.49)	0.140(0.35)	0.520(0.51)	0.340(0.48)	4.300(1.44)
6-year-olds	0.980(0.14)	0.900(0.03)	0.980(0.14)	0.850(0.36)	0.270(0.45)	0.640(0.49)	0.560(0.50)	5.290(1.16)
F(2, 123)	1.75	1.640	21.850	32.880	3.870	18.480	16.030	44.740
ηp^2^	0.028	0.026	0.262	0.348	0.059	0.231	0.207	0.421
*p*	0.178	0.198	<0.001	<0.001	0.230	<0.001	<0.001	<0.001

Note: ^1^ Total Score: Sum of 7 ToM tasks (range: 0–7), with 1 point for each correct task.

**Table 4:** Empathy ability mean, standard deviation, and analysis of variance (ANOVA).

Age Group	Emotional Empathy	Standard Deviation	Cognitive Empathy	Standard Deviation	Behavioral Empathy	Standard Deviation	Total Empathy Score	Standard Deviation
4-year-olds	0.800	14.14	8.733	4.35	5.366	4.81	15.600	15.21
5-year-olds	2.614	10.88	9.795	4.27	8.954	4.28	20.272	15.32
6-year-olds	6.346	10.39	9.019	4.27	7.961	3.87	21.519	13.88
Overall	3.722	11.68	9.222	4.28	7.69	4.44	19.674	14.78
ηp^2^	0.039		0.010		0.096		0.025	
F(2, 123)	1.595		0.645		6.533		1.595	
*p*	0.207		0.526		0.002		0.207	

**Table 5:** Correlation Analysis of Screen Exposure and ToM.

	Age	Gender	Only Child	Children’s Content	Average Daily Duration	Discussing Plot and Emotion
Different Desires	0.076	−0.070	−0.049	0.202 *	−0.287 **	−0.114
Different Beliefs	0.120	0.186 *	−0.035	−0.206 *	−0.073	0.156
Knowledge Access	0.407 **	0.229 *	0.070	0.019	0.156	−0.093
Content Error	0.518 **	0.166 +	−0.011	0.082	−0.238 **	0.161 +
Hidden Emotion	0.130	0.140	−0.089	−0.097	−0.051	0.157 +
Secondary Ignorance	0.442 ***	0.214 *	0.009	0.004	−0.037	0.089
Secondary False Belief	0.445 ***	0.198	0.079	0.161+	−0.127	0.069
Total Score of ToM	0.592 **	0.279 **	0.983	0.304	−0.202 *	0.202 *

Notes: *** Correlations were significant at the 0.001 confidence level (two-tailed). ** Correlations were significant at the 0.01 confidence level (two-tailed). * Correlations were significant at the 0.05 confidence level (two-tailed). + Correlations were marginally significant at the 0.06 confidence level (two-tailed). Spearman’s rank correlations were used for dichotomous variables (e.g., “Only Watching Children’s Content,” individual ToM tasks) and non-normal continuous variables. Pearson correlations were used for normally distributed continuous variables (e.g., age, total ToM score).

**Table 6:** Hierarchical regression analysis on the total score of ToM and screen exposure.

ToM	β	B	Standard Error	t	Significance
Step 1					
Age	0.571	1.105	0.136	8.152	<0.001
Gender	0.227	0.736	0.227	3.243	0.002
Adjusted R^2^	0.392 ***				
Step 2					
Daily Time	−0.14	−0.007	0.004	2.027	0.045
Only Watch Children’s Content	0.137	0.581	0.294	1.974	0.051
Adjusted ΔR^2^/Adjusted R^2^	0.026 */0.418 ***				
Step 3					
Discuss Plot	0.232	0.497	0.144	3.458	0.001
Adjusted ΔR^2^/Adjusted R^2^	0.049 **/0.467 ***				
F	22.862 ***				df = 2, 123

Notes: The Parental Mediation variable in the final model excludes the “co-viewing” item, as preliminary analyses showed it did not correlate with ToM or improve model fit. Future research should disentangle the distinct roles of passive co-viewing versus active, dialogic mediation, as these represent different parenting strategies with unique effects. *** Correlation is significant at the 0.001 level (two-tailed). ** Correlation is significant at the 0.01 level (two-tailed). * Correlation is significant at the 0.05 level (two-tailed).

**Table 7:** Correlation Analysis between Empathy Ability and Screen Exposure.

	Emotional Empathy	Cognitive Empathy	Behavioral Empathy	Total Empathy Score
Age	0.232 **	0.031	0.181 *	0.190 *
Gender	0.176 *	−0.092	−0.042	0.094
Only Child	−0.003	−0.022	−0.044	−0.007
Daily Time	−0.035	0.043	0.030	−0.010
Only Watch Children’s Content	−0.081	−0.059	0.025	−0.078
Parent–Child Discussion	0.078	−0.021	0.002	0.093

** Correlation is significant at the 0.01 level (two-tailed). * Correlation is significant at the 0.05 level (two-tailed). Note: Spearman’s rank correlations were used for dichotomous variables (e.g., “Only Watching Children’s Content”). Pearson correlations were used for continuous variables (e.g., age, Affective empathy score).

**Table 8:** Regression Analysis of Screen Exposure and Revised Composite Empathy Score.

Variables	B	Unstandardized Coefficients SE	Standardized Coefficient Beta	t	Significance
(Constant)	−3.25	10.50	-	−0.31	0.756
Age	2.85	1.50	0.175	1.90	0.062
Gender	1.50	2.50	0.055	0.60	0.550
Only Child	−0.25	3.00	−0.007	−0.08	0.936
Daily Average Time	0.005	0.040	0.010	0.125	0.901
Only Watch Children’s Content	−1.50	3.20	−0.040	−0.469	0.640
Discussion of Plot Emotions	1.80	1.65	0.095	1.091	0.278

Note: The revised composite empathy score includes only the reliable components (GEM Affective subscale, GEM Cognitive subscale, parent-reported empathic behaviors) to avoid measurement error from the unreliable GEM total score. None of the screen exposure variables significantly predicted the revised score (all *p* > 0.05).

## Data Availability

The original data supporting the findings of this study are available from the corresponding author upon reasonable request. Due to ethical restrictions and privacy considerations (to protect the anonymity of kindergarten participants and their families), the data are not publicly archived. Requests for data access may be directed to Yichen Wang (wang-yc22@mails.tsinghua.edu.cn), and all inquiries will be evaluated in accordance with institutional data sharing policies.
